# Supplementary material for: Acute effects of variable resistance training on force, velocity, and power measures: a systematic review and meta-analysis
Source: PeerJ. 2022 Aug 17;10:e13870. doi: 10.7717/peerj.13870 (PMC9392455; doi:10.7717/peerj.13870)
Supplement: Supplemental Information 4 [file peerj-10-13870-s004.docx]

1. **The rationale for conducting the systematic review**

The acute neuromuscular responses to utilizing elastic bands or chains during resistance training have been widely investigated. However, whether the effects of variable resistance training (VRT) on neuromuscular adaptations is better than that of constant resistance training (CRT) are still controversial. No systematic reviews to date have investigated this aspect. Hence, we used a meta-analytical approach to determine the differences between VRT and CRT controlling for the loading schemes, the contribution of variable resistance, and equipment on force, velocity, and power outcomes.

1. **The contribution that the systematic review makes to knowledge in light of previously published related reports, including other meta-analyses and systematic reviews**

To our knowledge, this is the first systematic review to provide evidence by investigating acute neuromuscular differences between VRT and CRT. The results revealed velocity and power benefit from the use of VRT. In addition, the neuromuscular responses to VRT differ across different loading schemes, variable resistance equipment, and contributions of variable resistance, which are important considerations for prescribing a VRT strategy. Therefore, different VRT strategies should be considered in context of an individual’s needs.
