# Supplementary figures and images for: Acute effects of variable resistance training on force, velocity, and power measures: a systematic review and meta-analysis
Source: PeerJ. 2022 Aug 17;10:e13870. doi: 10.7717/peerj.13870 (PMC9392455; doi:10.7717/peerj.13870)

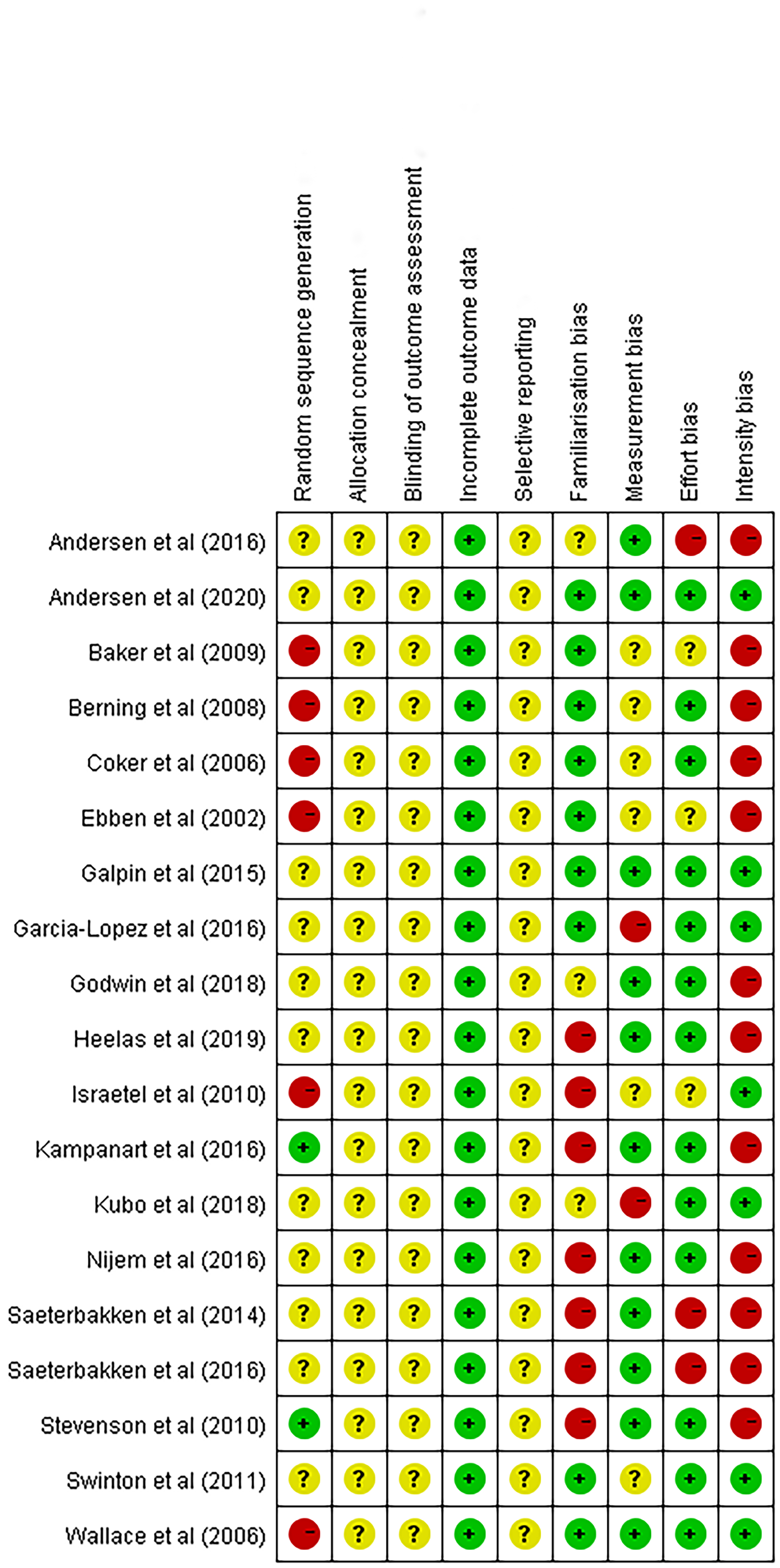

Supplement: Figure S1 [file peerj-10-13870-s005.png]
