# Supplementary material for: Acute effects of variable resistance training on force, velocity, and power measures: a systematic review and meta-analysis
Source: PeerJ. 2022 Aug 17;10:e13870. doi: 10.7717/peerj.13870 (PMC9392455; doi:10.7717/peerj.13870)
Supplement: Table S1 [file peerj-10-13870-s006.docx]

**Supplementary Material**

**Table S1.** Search terms

| **PubMed:**  (“elastic band”[All fields] OR “rubber band”[All fields] OR “thera-band”[All fields] OR “elastic tubing”[All fields] OR “chain”[All fields]) AND ((“variable resistance”[All fields] OR “accommodating resistance”[All fields] OR “resistance training”[All fields] OR “free weight”[All fields] OR “back squat”[All fields] OR “bench press”[All fields] OR “deadlift”[All fields] OR “weightlifting”[All fields]) AND (“kinetic”[All fields] OR “kinematic”[All fields] OR “force”[All fields] OR “power”[All fields] OR “velocity”[All fields]) |
| --- |
| **Web of Science:**  #1. TS = (“elastic band” OR “rubber band” OR “thera-band” OR “elastic tubing” OR chain)  #2. TS = (“variable resistance” OR “accommodating resistance” OR “resistance training” OR “free weight” OR “back squat” OR “bench press” OR deadlift OR weightlifting)  #3. TS = (kinetic OR kinematic OR force OR power OR velocity)  #. #1 AND #2 AND #3 |
| **SPORTDiscus:**  (“elastic band” OR “rubber band” OR “thera-band” OR “elastic tubing” OR chain) AND (“variable resistance” OR “accommodating resistance” OR “resistance training” OR “free weight” OR “back squat” OR “bench press” OR deadlift OR weightlifting) AND (kinetic OR kinematic OR force OR power OR velocity) |
